# A Graduate Medical Education Curriculum to Introduce the Concept of Cancer Survivorship

**DOI:** 10.15766/mep_2374-8265.10673

**Published:** 2018-01-25

**Authors:** Regina A. Jacob, Vanneta Hyatt

**Affiliations:** 1 Assistant Professor, Section of General Internal Medicine, Lewis Katz School of Medicine at Temple University

**Keywords:** Oncology, Workshop, Primary Care, Cancer Survivorship, Ambulatory Curriculum

## Abstract

**Introduction:**

The growing number of cancer survivors has expanded the need for physicians familiar with survivorship care outside the subspecialty of oncology. However, primary care providers have historically not been comfortable managing the growing number of cancer survivors and their long-term sequela. To date, there is no current ambulatory curriculum designed as a workshop to discuss general concepts of cancer survivorship with internal medicine resident physicians.

**Methods:**

This was a 3-day workshop series given over 5 weeks. Residents were given a simulated case following a geriatric breast cancer survivor. Session 1 consisted of creating a survivorship care plan and discussing secondary cancer screening. Session 2 included a discussion on primary tumor recurrence and short-term side effects of cancer treatments. Finally, Session 3 incorporated mental health adjustment and long-term side effects of various cancer treatments. Knowledge and attitude assessments were administered during Sessions 1 and 3.

**Results:**

Eighty-seven residents participated, with 59 completing the survey on Session 1, and 36 completing the same survey on Session 3. Prior to the curriculum, two residents reported comfort in creating a survivorship care plan compared to 23 after the curriculum. Similarly, comfort in screening for excess mortality increased from three residents to 19. General knowledge of common side effects from cancer treatment modalities was well known prior to the curriculum.

**Discussion:**

Survivorship care heavily incorporates the basics of primary care. This curriculum successfully raised awareness among resident physicians regarding survivorship terminology, which, in turn, improved their comfort in the long-term management of the cancer survivor.

## Educational Objectives

By the end of this module, the learner will be able to:

1. Explain the basic terminology used in survivorship care (i.e., background mortality, excess mortality, secondary malignancies, and primary recurrence).
2. Create an individualized survivorship care plan for the adult breast cancer survivor.
3. Identify appropriate screening tests for long-term surveillance of the breast cancer survivor.
4. Identify common short-term side effects of various cancer treatment modalities.
5. Classify physical symptoms that could be early indications of primary recurrence or secondary malignancies.
6. Identify appropriate diagnostic tests for concerning physical symptoms in the adult breast cancer survivor.
7. Differentiate chronic morbidities that arise from various cancer treatments.
8. Address mental health concerns in the cancer survivor.
9. Classify physical symptoms that could be early indications of long-term side effects of cancer treatments and excess mortality.

## Introduction

The growing number of cancer survivors has expanded the need for physicians familiar with survivorship care outside the subspecialty of oncology.^1,2^ In response to the 2015 initiative by the Commission on Cancer to incorporate survivorship care planning in order to receive accreditation,^3^ institutions have pushed for the adaptation of survivorship care into routine oncological care. Because cancer survival has improved dramatically, the full care of the cancer survivor—screening for secondary cancers, evaluating for cancer recurrence, managing chronic morbidities from cancer treatments, and addressing mental health adjustment—requires the involvement of primary care physicians and other subspecialists outside of oncology,^4,5^ in alignment with the established guidelines distributed by various cancer agencies. However, the level of comfort and confidence among primary care physicians with regards to managing the long-term care of cancer survivors is low.^6^ The largest barrier to survivorship care from the perspective of primary care physicians is the transition of care from oncology back to primary care, and the uncertainty involved in long-term surveillance.^6^

The following curriculum was designed to introduce the concept of survivorship care, and provides specific terminology to the existing outpatient graduate medical education curriculum. The resource created for distribution was designed to follow a geriatric breast cancer survivor through three simulated primary care visits. A workshop format was used to implement the designed curriculum, consisting of three 50-minute group sessions per week for 5 weeks. The specific learning objectives were tailored to the long-term outpatient management of the adult breast cancer survivor. The survivorship curriculum was taught along with a geriatrics component, but the focus of this module is the new topic of survivorship.

The target audience for this workshop includes first- through third-year internal medicine residents in the ambulatory setting. There was extremely limited prior exposure to the issues of survivorship care among this group of learners. Curriculum facilitators were primary care physicians without specific training in oncology.

## Methods

The workshop was conducted during the outpatient ambulatory block in October and November of 2016. There were 99 categorical internal medicine residents who rotated through the sessions. Occasionally, third- and fourth-year medical students were present, but this curriculum was designed specifically for graduate medical education. There were 87 residents who participated in this curriculum, as six residents were in the primary care track and six were on vacation during the workshop period.

The simulated case (Appendix A) with specific learning objectives was designed to follow a geriatric breast cancer survivor in outpatient primary care. An accompanying facilitator manual (Appendix B) was included to assist users with the implementation of the case.

Learners were given the review article “In the Clinic: Care of the Adult Cancer Survivor” prior to the start of the first session.^2^ Since little is known among internal medicine residents about survivorship care, the article provided background knowledge for learners and served as a reference guide throughout the workshop. The discussion and determination of appropriate surveillance and complete survivorship care was based on the American Cancer Society/American Society of Clinical Oncology (ASCO) Breast Cancer Survivorship Care Guideline.^7^ The learners were also given a multiple-choice questionnaire (Appendix C; answer key Appendix D) on Sessions 1 and 3 of the workshop to assess their knowledge initially, and to track improvement. The survey consisted of six questions, with four focused on specific survivorship terminology and common side effects of cancer treatment, and two that assessed physician comfort in managing the care of the cancer survivor.

### Session 1 Structure (50 minutes)

• Questionnaire.
• Survivorship Care Plan.
• Breast Cancer Surveillance.
• Secondary Cancer Screening.

The multiple-choice questionnaire was administered to individual learners during the first 10 minutes of the session. Learners were divided into three to four groups by the facilitator depending on attendance, and they were grouped with an even distribution of postgraduate year representation. Session 1 learning objectives consisted of creating a survivorship care plan (Appendix E; completed example, Appendix F) and discussing secondary cancer screening. The ASCO Treatment Summary and Survivorship Care Plan^8^ was used as the template for learners. The learners were given 15 minutes to discuss and complete a care plan in groups. The small groups then reconvened as one large group for the remainder of the session to discuss the care plans and review answers to the learning objectives.

### Session 2 Structure (50 minutes)

• Primary Tumor Recurrence.
• Long-Term Side Effects of Cancer Treatments (Excess Mortality).

During this session, the learners reconvened into small groups to discuss the case progression. During this time the residents learned that the patient returned for follow-up with a chief complaint of urinary incontinence and falls. Learners were expected to discuss side effects of various cancer treatments, and how a cancer history could contribute to these physical symptoms. They were also expected to correlate physical symptoms with early signs of primary recurrence or secondary malignancy.

### Session 3 Structure (50 minutes)

• Mental Health Assessment.
• Late Recurrence.
• Questionnaire.

The third and final session was conducted in a similar format to the previous session. The learners assembled into their respective small groups and they were given 15 minutes to review the case progression and discuss the associated learning objectives for this session. During the session they learned that the case patient returned for her third visit with complaints of memory difficulties and sleep disturbances. Learners discussed mental health adjustment and long-term side effects of various cancer treatment modalities. The remaining 5–10 minutes allowed the learners to complete the questionnaire previously administered during Session 1.

## Results

Over the course of 5 weeks, 87 internal medicine residents rotated through the outpatient curriculum. Of these, 59 (67.80%) completed the introduction survey on Session 1, and 36 (41.38%) completed the same exit survey on Session 3. Overall, resident physicians reported an improvement in their comfort in managing care of the cancer survivor.

**Figure 1.**
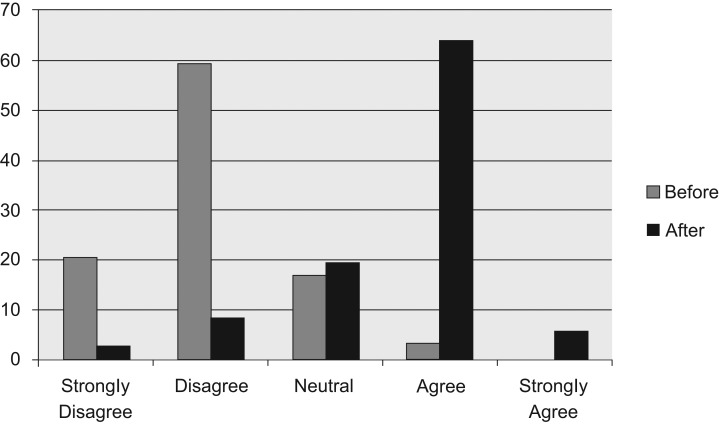
Percentage of learners who felt competent in knowing how to find or create a survivorship care plan, before and after the curriculum.

Prior to the curriculum, the majority of resident physicians stated that they either disagreed (59.32%) or strongly disagreed (20.34%) with the question assessing how competent they felt in their knowledge of how to find or create a survivorship care plan. After the curriculum, the majority of resident physicians stated that they either agreed (63.89%) or strongly agreed (5.56%) when asked the same question (Figure 1).

**Figure 2.**
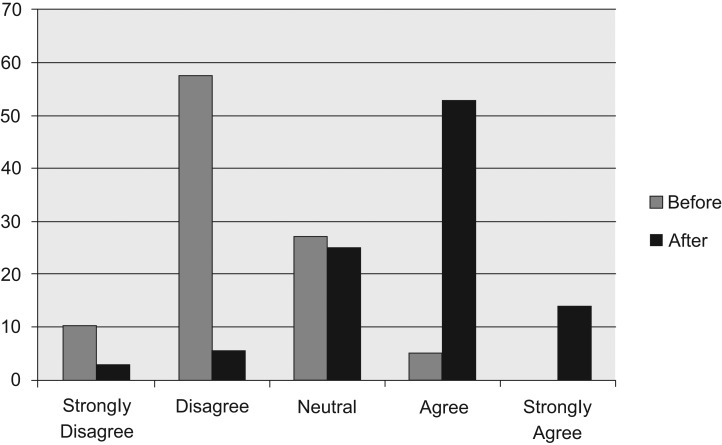
Percentage of learners who felt comfortable in screening for excess mortality in cancer survivors, before and after the curriculum.

Resident physicians also reported an improvement in their comfort with screening for excess mortality. Prior to the curriculum, the majority of resident physicians stated that they either disagreed (57.63%) or strongly disagreed (10.17%) when asked if they were comfortable in managing excess mortality in the cancer survivor. Following the curriculum, resident physicians reported that they were much more comfortable in the management of excess mortality in the cancer survivor, with 52.78% agreeing and 13.89% strongly agreeing with this statement (Figure 2).

With regards to general knowledge, there was minimal change before and after the curriculum. When asked about the common long-term side effects of chest radiation, 100% answered correctly before the curriculum, compared to 94.44% who answered correctly after the curriculum. Regarding knowledge of the growing number of cancer survivors in the country, 98.10% answered correctly prior to the curriculum, while 100% answered correctly after the curriculum. Both questions were answered correctly by an overwhelming majority in the precurriculum and the postcurriculum assessments.

## Discussion

The curriculum was successful in alerting resident physicians to the basics of survivorship care. Per responses to the questionnaire, resident physicians were aware of the growing numbers of cancer survivors and their health care needs. Small-group discussions indicated that they were not only cognizant of long-term care needs, but were also aware of the importance of primary care physicians in the surveillance setting. Facilitated discussions also emphasized that survivorship care required a collaborative approach that involved providers outside of primary care and oncology, including nutrition, subspecialty providers, palliative care, behavioral health, and physical therapy. Resident physicians reported unfamiliarity with who was responsible for specific surveillance testing—oncologists or primary care physicians—but they did recognize the importance of long-term surveillance and discussed the need for collaborative care.

With regards to basic knowledge of side effects from cancer treatments, resident physicians were already aware of common side effects of various treatment modalities prior to the curriculum. This was reflected both in the small-group discussions and in the answers to the knowledge assessment questionnaire administered prior to the start of the workshop series. However, it became apparent during the course of the curriculum that resident physicians lacked awareness of the specific terminology used in survivorship care. For example, they were well aware of the cardio-pulmonary complications of radiation therapy, but were unaware that this was referred to as “excess mortality.” Additionally, resident physicians were unaware of the existence of survivorship care plans. Exposing them to survivorship care plans, and engaging them in the exercise of creating a care plan with a simulated case patient, increased their self-reported comfort in being able to provide long-term survivorship care. They reported that the creation of these survivorship care plans or knowing where to find it was the most useful aspect of this curriculum.

### Limitations

One limitation to analyzing the overall success of the curriculum was inconsistent attendance to morning conferences due to other requirements. Additionally, there was a large variance in resident participation on each day. The residents who answered the survey on Session 1 might not have answered again on Session 3 and vice versa. Regardless, the overwhelming trend is that the workshop allowed participants to feel more comfortable in managing long-term care of the cancer survivor.

A second limitation is whether or not learners will recall this exercise in the future. Learners were already well aware of the common side effects of various cancer treatments and already knew the definition of “cancer survivor,” as demonstrated by the ceiling effect seen in the knowledge assessment. This aptitude simply needed to be brought to the forefront of awareness, which was facilitated by the workshop discussions. Learners were made aware of specific terminology involved in survivorship care which seemed to improve overall comfort. As such, longstanding retention might be sufficient, though not specifically measured.

### Lessons Learned and Future Implications

This curriculum was administered alongside a geriatrics team-based learning workshop. As such, the discussions particular to the survivorship component of the curriculum were more heavily addressed in the first session. Future curricula could be arranged differently so that there is an even distribution of time dedicated to individual topics in survivorship care. Furthermore, a survivorship curriculum could exist on its own given the breadth of topics available for discussion.

As this was the first exposure to survivorship care for the majority of the participating learners, comfort in using specific tools such as the survivorship care plan and the ACS/ASCO guidelines could not be thoroughly assessed in a classroom setting. Repeating the curriculum—perhaps using a different simulated case with a different cancer diagnosis—after sufficient time to allow for residents to implement these specific skills in their ambulatory clinics, could show increased levels of comfort in survivorship care.

Since survivorship care models the basics of primary care, this curriculum could be structured to incorporate the traditional ambulatory care milestones and can be another useful tool for evaluation. Finally, multiple-choice questions could be rewritten to conform to USMLE standards and could also assess more challenging survivorship issues since learners at this level already have basic aptitude.

### Conclusions

Despite having a high degree of knowledge with regards to common survivorship issues (e.g., osteoporosis, cardiopulmonary complications, recurrence, secondary cancers, and mental health distress) resident physicians still reported that they were uncomfortable in their ability to provide care for the cancer survivor. After the curriculum, however, resident physicians reported an improvement in their comfort in caring for the cancer survivor while their overall knowledge of survivorship issues remained unchanged but sufficient. Given this finding, it may be said that this curriculum, although improving knowledge of the transition from oncology back to primary care and outlining roles for providers in the future, raises awareness of terminology that may be the key to familiarizing resident physicians on the long term management of surivors. The understanding of this terminology may be a key factor in physician comfort in caring for the cancer survivor.

## Appendices

A. Survivorship Case.docx
B. Facilitator Manual.docx
C. Pre- and Posttest.docx
D. Pre- and Posttest with Answers.docx
E. ASCO Survivorship Care Plan Blank.docx
F. ASCO Survivorship Care Plan Bonnie Olden.docx All appendices are peer reviewed as integral parts of the Original Publication.
