# Supplementary material for: A Graduate Medical Education Curriculum to Introduce the Concept of Cancer Survivorship
Source: MedEdPORTAL. 2018 Jan 25;14:10673. doi: 10.15766/mep_2374-8265.10673 (PMC6342428; doi:10.15766/mep_2374-8265.10673)
Supplement: Supplementary file 1 — A. Survivorship Case.docx B. Facilitator Manual.docx C. Pre- and Posttest.docx D. Pre- and Posttest with Answers.docx E. ASCO Survivorship Care Plan Blank.docx F. ASCO Survivorship Care Plan Bonnie Olden.docx [file mep-14-10673-s001.zip › A._Survivorship_Case.docx]

**Case for Sessions 1-3**

**SESSION 1**

Ms. Bonnie Olden (DOB 7/20/1944) presents to the primary care office to establish care. Six years ago, she was diagnosed with breast cancer. Her tumor was 2.5 cm in size at the time of diagnosis and the pathology revealed high grade characteristics. She underwent mastectomy and axillary lymph node dissection which showed 3/12 positive lymph nodes. Tumor was ER positive, and HER-2 negative.

Diagnosis: Stage III breast adenocarcinoma.

**Treatment included:**

Surgical Resection: LEFT mastectomy and Lymph Node Dissection on 4/29/2010 (Dr. Elaine Cutting)

Radiation therapy: Received radiation therapy to the LEFT breast. Treatment ended 8/10/2010 (Dr. Ex Ray)

Chemotherapy: received 4 cycles of Doxorubicin, Cyclophosphamide, and Paclitaxel, completed 7/10/2010 (Dr. Ivy Infu Sion).

She is currently on Arimidex and has been in remission for the last 5 years. She has been discharged from her oncologist and is currently here to establish care with a primary care physician. Her other past medical history includes hypertension, depression, coronary artery disease, osteoarthritis, hyperlipidemia, diabetes mellitus, and seasonal allergies.

Surgery:

- C-section x 2
- LEFT Breast surgery
- LEFT Axillary lymph node dissection

Family History:

- Noncontributory

Social History:

- Tobacco – smoked 2 packs per day for 40 years prior to her cancer diagnosis; now smokes an occasional cigarette
- ETOH – 1-2 glasses of wine a week
- No other illicit substance use
- Lives alone in her own house, but daughter lives nearby and accompanies her to appointments.
- Independent with activities of daily living (ADL)

She wants to know how often she should get mammograms and whether or not she should be getting other cancer screenings. She is also worried about infrequent visits with her oncologist and wants to know plans for follow up, particularly regarding her breast cancer. She reports persistent fatigue since the diagnosis 6 years ago.

Today she brings with her a medication list as well as her medication bottles which corroborate accordingly.

**MEDICATIONS:**

Aspirin 81mg daily

Metformin 500mg twice daily

Glipizide 5mg twice daily

Atorvastatin 20mg daily

Naproxen 375mg as needed

Oxybutynin XL 5mg daily

Paroxetine 20mg daily

Lisinopril 40mg daily

Metoprolol XL 50mg daily

HCTZ 25mg daily

Benadryl 25mg as needed

Coenzyme Q daily

Fluticasone nasal spray daily or as needed

Omeprazole 20mg daily

Session 1 Learning Objectives:

1. **Make a Survivorship Care Plan**
   1. **List major concerns in long term follow up because of each treatment modality listed**
      1. **Radiation therapy**
      2. **Chemotherapy**
         1. **Doxorubicin**
         2. **Cyclophosphamide**
         3. **Paclitaxel**
         4. **Arimidex**
   2. **Discuss the appropriate surveillance plan for breast cancer.**
   3. **Discuss the plan for secondary cancer screening.**
2. *Discuss Polypharmacy and how to screen for it.*

Session 1 Questions:

1. **How does a prior cancer diagnosis change cancer screenings?**
2. **When should we be screening for other cancers?**
3. **Are there other cancers we should be considering given her particular breast cancer treatment history?**
4. **Are there other co-morbidities we should be screening for because of her cancer history?**
5. *Polypharmacy: what are some ways to reduce medication errors? What are some factors that can affect the appropriate prescription of medications?*
6. *What are Beer’s criteria?*

**SESSION 2**

Ms. Olden returns 6 weeks after her initial visit. She reports one fall 2 weeks ago. She denies any chest pain or palpitations before this fall. She denies LOC or head injury. She states she was going to answer the phone and “next thing I knew I was on the floor.”

Since the fall her right knee has been aching. She reports some swelling in the right knee after the fall. She used *AsperCreme* and rubbing alcohol on her knee which helped some, but the pain persists. She wants to know whether there is something to help with the pain.

Additionally, she reports some urinary incontinence. She states she was on a medication for overactive bladder in the past but does not know whether it is working well.

Session 2 Learning Objectives:

1. *Fall Prevention in Aging Adults*
2. *Urinary incontinence*
3. **Identify how chemotherapeutic agents and radiation therapy can contribute to her recent falls.**
   1. **Discuss additional testing that could be warranted.**
4. **Identify how chemotherapeutic agents and radiation therapy can contribute to urinary incontinence.**
   1. **Discuss additional testing that could be warranted.**

Session 2 Questions:

1. *Falls are generally multifactorial. What are some of the common risk factors for falls in the elderly? Intrinsic? Extrinsic? Environmental? What are some medications that are commonly associated with falls?*
2. *What diagnostic tests (labs/imaging) would you order to rule out potential etiologies that might contribute to falls? What geriatric mobility assessment tool is available to evaluate a patient’s risk of falls?*
3. **How does her cancer history potentially contribute to her fall?**
   1. **Are there any particular concerns with chemotherapeutic agents or radiation therapy she received to treat her breast cancer?**
   2. **Would you do any additional testing? If so, why?**
4. *How would you characterize urinary incontinence? What are the 4 different types of urinary incontinence?*
5. **How would her cancer history influence the evaluation of urinary incontinence?**
   1. **Are there any particular concerns with chemotherapeutic agents or radiation therapy she received to treat her breast cancer?**
   2. **Would you do any additional testing? If so, why?**
6. *What is the recommended first line therapy for stress incontinence? Urge incontinence? Second line therapy for urge incontinence?*

**SESSION 3**

CC: memory problems and one more fall

Ms. Olden returns 4 months later with her daughter to the visit today. Her daughter reports that her mother seems to be getting more forgetful. Symptoms began approximately 1 year ago however has worsened since then. The patient has had multiple conversations regarding topics previously discussed but with each subsequent discussion, appears as if she is hearing it for the first time. She has no difficulty with her ADLs or traveling however has forgotten to pay a few bills on time. She reports she still misses her husband who passed away 3 years ago. She reports some sleep disturbances for which she has been using Ambien.

Session 3 Learning Objectives:

1. **Discuss the work up and evaluation of depression and cognitive impairment.**
   1. **Discuss the available tools to assist in delineating your diagnosis.**
2. **How might her cancer history and treatment contribute to impaired memory?**
   1. **Would you require any additional testing?**

Session 3 Questions:

1. **How would you evaluate her symptoms?**
   1. **How can you evaluate for depression?**
   2. **How can you evaluate for Mild Cognitive Impairment?**
2. **How would her cancer history potentially contribute to her current symptoms of forgetfulness? Discuss any tests you would order.**
   1. **How can chemotherapeutic agents or radiation therapies contribute to memory loss? Discuss any tests you would order.**
3. *What are some other components of the geriatric assessment?*
4. *What if our patient was 87 – what are some other key components you would need for evaluation? Are there instruments/scales available to assist you?*
