# Supplementary material for: A Graduate Medical Education Curriculum to Introduce the Concept of Cancer Survivorship
Source: MedEdPORTAL. 2018 Jan 25;14:10673. doi: 10.15766/mep_2374-8265.10673 (PMC6342428; doi:10.15766/mep_2374-8265.10673)
Supplement: Supplementary file 1 — A. Survivorship Case.docx B. Facilitator Manual.docx C. Pre- and Posttest.docx D. Pre- and Posttest with Answers.docx E. ASCO Survivorship Care Plan Blank.docx F. ASCO Survivorship Care Plan Bonnie Olden.docx [file mep-14-10673-s001.zip › B._Facilitator_Manual.docx]

**FACILITATOR’S MANUAL**

This curriculum is designed for internal medicine residents but can be used to discuss the relevant aspects of survivorship care in other subspecialties and practices. The facilitators for this curriculum are general internists since many of the topics are pertinent to primary care. If facilitators have a background in cancer survivorship, it is helpful but not required.

For successful implementation of this curriculum, facilitators should be familiar with the ASCO Survivorship Care Plan and be able to complete a care plan using relevant information provided in the case. Additionally, facilitators should be familiar with the topics discussed in the pre-reading article assigned to the learners—*Care of the Adult Cancer Survivor*^1^*.* For reference, the survivorship guidelines used to develop this curriculum are derived from *American Cancer Society/American Society of Clinical Oncology Breast Cancer Survivorship Care Guideline*^2^*.*

**- SESSION 1 -**

The session begins with the assumption that all learners and facilitators have read *Care of the Adult Cancer Survivor* and are familiar with the topics discussed in that article. Initiate the session by having learners read the case presentation for Ms. Bonnie Olden.

**Session 1 Learning Objectives:**

1. Make a Survivorship Care Plan (15 minutes) – Learners should fill out the ASCO survivorship care plan using the information given in the case presentation for Ms. Bonnie Olden. Facilitators may choose to divide the learners into groups to complete this task in order to encourage discussion and collaboration. After the allotted time is completed, review the care plan as a larger group, focusing the discussion on appropriate follow up care. (See completed ASCO Survivorship Care Plan for Ms. Bonnie Olden).
   1. List the major concerns in long term follow up which result from each treatment modality
      1. Radiation therapy: follow-up and relevant screening is based on location of radiation. In this case, the patient received chest radiation.
         1. Thyroid Dysfunction – long term follow-up should include screening for relevant symptoms in the history including fatigue, hair/skin/nail changes, and changes in bowel habits.
            1. Screening TSH might be warranted on a regular basis
         2. Restrictive Cardiomyopathy – long term follow-up should include screening for relevant symptoms in the history including fatigue, chest pain, lower extremity edema, dyspnea on exertion, palpitations, and paradoxical nocturnal dyspnea.
            1. Currently there is no evidence for annual echocardiography or electrocardiogram in asymptomatic patients. If symptoms develop, appropriate imaging and a referral to the appropriate specialist may be warranted.
         3. Radiation Pneumonitis – long term follow-up should include screening for relevant symptoms in the history including fatigue, dyspnea on exertion, and cough.
            1. Currently there is no evidence for annual pulmonary function tests or radiological studies in asymptomatic patients. If symptoms develop, appropriate imaging and a referral to the appropriate specialist may be warranted.
         4. Radiation Esophagitis – long term follow-up should include screening for relevant symptoms in the history including chest pain, odynophagia, dysphagia, excessing belching, or abdominal pain.
            1. Currently there is no evidence for annual endoscopy or radiological studies in asymptomatic patients. If symptoms develop, referral to the appropriate specialist for endoscopy may be warranted.
      2. Chemotherapy
         1. Doxorubicin – long term concern is primarily dilated cardiomyopathy. Follow-up should include screening for relevant symptoms in the history including fatigue, chest pain, lower extremity edema, dyspnea on exertion, palpitations, and paradoxical nocturnal dyspnea.
            1. Currently there is no evidence for annual echocardiography or electrocardiogram in asymptomatic patients. If symptoms develop, appropriate imaging and a referral to the appropriate specialist may be warranted.
         2. Cyclophosphamide— long term concern is secondary malignancy, particularly leukemia and bladder carcinoma.
            1. Annual CBC might be warranted to evaluate for developing pathology.
            2. There is no evidence for annual urinalysis, but should a patient develop hematuria, referral to the appropriate specialist for cystoscopy is warranted.
            3. There is no evidence for annual radiological studies in asymptomatic patients to screen for pneumonitis.
         3. Paclitaxel – most common long term side effect is peripheral neuropathy.
            1. There is no screening for peripheral neuropathy and treatment is primarily symptomatic with acetaminophen, non-steroidal therapy, duloxetine, acupuncture, etc .
         4. Anastrozole – most common and concerning long term side effects include coronary artery disease, venous thromboembolism, cognitive dysfunction, osteoporosis, and anxiety.
            1. Screening for these issues consists of good history taking and asking about relevant symptoms.
            2. Screening for coronary artery disease follows the current USPSTF recommendations:

The USPSTF recommends annual lipid panel for men>35 years and women>45 years; and statin therapy should be initiated when warranted.

The USPSTF recommends screening for abnormal blood glucose as part of cardiovascular risk assessment in adults aged 40 to 70 years who are overweight or obese.

The USPSTF recommends screening for high blood pressure in adults aged 18 years or older. Antihypertensive therapy should be initiated when warranted.

Counseling on smoking cessation should be provided.

Counseling on weight management should be provided.

Counseling on stress management should be provided.

- - - - 1. If patient reports symptoms concerning for cognitive decline, contacting the patient’s primary oncologist is warranted as duration of therapy might need to be discussed and determined.
        2. ACS/ASCO recommends baseline DEXA scan to evaluate for osteoporosis in all post-menopausal women on an aromatase-inhibitor. Repeat DEXA scans should be done every 2 years while on an aromatase inhibitor.
  1. Breast Cancer Surveillance – surveillance is usually specific to the individual patient and their provider. Contacting the primary oncologist to determine frequency of follow-up imaging might be warranted if the management plan is unclear.
     - 1. Annual mammography is warranted unless more frequent testing is specified.
          1. Follow up with radiation oncologist and/or breast surgeon is typically on a warranted basis unless otherwise specified.
          2. There is no evidence for breast MRI surveillance unless the patient meets high-risk criteria for breast cancer surveillance. If this is not specified, and there is concern that the patient might meet high-risk criteria, the primary care provider might need to contact the primary oncologist to discuss appropriate surveillance.
       2. Signs and symptoms of recurrence: the primary care physician can offer counseling to survivors regarding the signs and symptoms that are concerning for recurrence and encourage patients to alert providers should anything arise.
  2. Secondary Cancer Screening – age-appropriate cancer screening is recommended.
     - 1. The USPSTF recommends annual screening for lung cancer with low-dose computed tomography in adults aged 55 to 80 years who have a 30 pack-year smoking history and currently smoke or have quit within the past 15 years.
       2. The USPSTF recommends screening for cervical cancer in women age 21 to 65 years with Pap smear every 3 years or, for women age 30 to 65 years who want to lengthen the screening interval, screening with a combination of cytology and human papillomavirus (HPV) testing every 5 years.
       3. The USPSTF recommends screening for colorectal cancer using fecal occult blood testing, sigmoidoscopy, or colonoscopy in adults, beginning at age 50 years and continuing until age 75 years.

**Session 1 Questions:**

1. **How does a prior cancer diagnosis change cancer screenings?**
   1. Facilitators can encourage discussion regarding Ms. Olden’s specific risks based on her age and her current habits. Learners will be able to recognize that cancer screenings in a cancer survivor are overall, no different from the general population. Age-appropriate and risk-appropriate cancer screenings are recommended.
2. **When should we be screening for other cancers?**
   1. The USPSTF recommends annual screening for lung cancer with low-dose computed tomography in adults aged 55 to 80 years who have a 30 pack-year smoking history and currently smoke or have quit within the past 15 years.
   2. The USPSTF recommends screening for cervical cancer in women age 21 to 65 years with Pap smear every 3 years or, for women age 30 to 65 years who want to lengthen the screening interval, screening with a combination of cytology and human papillomavirus (HPV) testing every 5 years.
   3. The USPSTF recommends screening for colorectal cancer using fecal occult blood testing, sigmoidoscopy, or colonoscopy in adults, beginning at age 50 years and continuing until age 75 years.
   4. There is no screening for endometrial cancers, but providers can educate patients regarding post-menopausal vaginal bleeding symptoms which should be reported immediately to health care providers so that further investigation is initiated.
3. **Are there other cancers we should be considering given her particular breast cancer treatment history?**
   1. Bladder Cancer: there are no current recommendations on screening for bladder cancer, but if a patient presents with hematuria or hematuria is an incidental finding, providers should consider the possibility of bladder cancer given cyclophosphamide therapy.
   2. Leukemia: an annual CBC should be monitored, particularly considering cyclophosphamide therapy.
   3. Thyroid Cancer: this is rare but can happen with a history of chest radiation. Thyroiditis is more common which is why an annual TSH should be considered for anyone with a history of chest radiation.
   4. Esophageal cancer: given the history of chest radiation, if the patient reports new symptoms of reflux, a prompt referral to the appropriate specialty for endoscopy and evaluation is recommended.
4. **Are there other co-morbidities we should be screening for because of her cancer history?**

Facilitators can encourage discussion with learners regarding the most common co-morbidities. Learners will be able to recognize that the co-morbidities of cancer survivors are, overall, no different from the general population. Age-appropriate and risk-appropriate screenings are recommended.

- 1. Mental Health and Well-being
  2. Obesity
  3. Hypertension
  4. Hyperlipidemia
  5. Tobacco Use
  6. Diabetes
  7. Osteoporosis

**-SESSION 2-**

**Learning Objectives:**

1. Identify how chemotherapeutic agents and radiation therapy can contribute to her recent falls.
   1. Discuss additional testing that could be warranted.
2. Identify how chemotherapeutic agents and radiation therapy can contribute to urinary incontinence.
   1. Discuss additional testing that could be warranted.

**Session 2 Questions:**

1. **How does her cancer history potentially contribute to her fall?**
   1. **Are there any particular concerns with chemotherapeutic agents or radiation therapy she received to treat her breast cancer?**
      1. Brain metastasis and neuropathy are the most significant concerns.
         1. Paclitaxel can contribute to neuropathy
      2. Would consider whether she is having arrhythmias given history of doxorubicin therapy.
      3. Would consider thyroid dysfunction given history of radiation therapy.
   2. **Would you do any additional testing? If so, why?**
      1. Consider a brain MRI to search for metastatic disease.
      2. Would check a CBC given history of cyclophosphamide and TSH given history of chest radiation
      3. Electrocardiogram given history of doxorubicin and to evaluate for arrhythmia, +/- echocardiogram if there are symptoms or signs of cardiomyopathy from history and physical exam.
      4. Would also consider Doppler ultrasound of the lower extremities +/- CT chest if history indicates possible venous thromboembolism given anastrozole and cigarette use.
2. **How would her cancer history influence the evaluation of urinary incontinence?**
   1. **Are there any particular concerns with chemotherapeutic agents or radiation therapy she received to treat her breast cancer?**
      1. Based on history of chest radiation, consider thyroid dysfunction
      2. Given history of cyclophosphamide therapy, consider secondary bladder cancer or cystitis.
   2. **Would you do any additional testing? If so, why?**
      1. Would check a TSH given history of chest radiation.
      2. Would check BMP and a urinalysis considering history of cyclophosphamide.

**-SESSION 3-**

**Learning Objectives:**

1. Discuss the work up and evaluation of depression and cognitive impairment.
   1. Discuss the available tools to assist in delineating your diagnosis.
2. How might her cancer history and treatment contribute to impaired memory?
   1. Would you require any additional testing?

**Session 3 Questions:**

1. **How would you evaluate her symptoms?**
   1. **How can you evaluate for depression?**
      1. The diagnosis of clinical depression is based on patient history and exclusion of alternative diagnoses. The evaluation must determine whether the patient meets established criteria for major depression, dysthymia, or a different psychiatric condition. The assessment should also include screening for substance abuse. Depressed mood and anhedonia are cardinal symptoms, and the presence of either is highly sensitive but not specific for major depression. Using a two-item questionnaire, i.e. PHQ-2, which assesses for the presence of depressed mood or anhedonia is a quick way to screen for depression. If either depressed mood or anhedonia is present, further inquiry or employing a second tool to diagnose depression should be pursued, i.e. PHQ-9.
   2. **How can you evaluate for Mild Cognitive Impairment?**
      1. Mild cognitive impairment is a cognitive state between normal aging and dementia characterized by a decline in cognitive functioning that is greater than what is expected with normal aging but has not resulted in significant functional disability. For most patients, the onset is insidious, and for some, the course may be progressive. 10% to 15% of patients with mild cognitive impairment transition to dementia per year, compared with 1% to 2% per year of the general population. The Montreal Cognitive Assessment is a screening tool that is more sensitive than the Mini–Mental State Examination in the detection of mild cognitive impairment because it has more cognitively challenging tests of memory/recall and executive function. Scoring lower than 26/30 generally suggests cognitive impairment, especially in patients with 16 years of formal education. In clinical practice, a careful history and results of a standard mental examination are often sufficient to make a diagnosis of mild cognitive impairment, and extensive cognitive testing is not routinely required. Occasionally, a formal battery of neuropsychological testing beyond the standard mental examination is needed to distinguish particularly mild cases of cognitive impairment from normal aging.
2. **How would her cancer history potentially contribute to her current symptoms of forgetfulness? Discuss any tests you would order.**
   - 1. Metastatic disease is always a concern with a cancer history
        1. Would consider CT/MRI head to evaluate for metastatic disease.
     2. Mental health assessments are recommended in all cancer survivors because the risk of depression, adjustment disorder or PTSD symptoms is elevated.
3. **How can chemotherapeutic agents or radiation therapies contribute to memory loss? Discuss any tests you would order.**
4. Anastrozole therapy can result in cognitive dysfunction.
   - - 1. If clinical suspicion of a drug side effect is high, this should encourage a discussion with the primary oncologist regarding duration of therapy and risks/benefits of continuing prolonged hormone therapy after 5 years.
5. Would screen for thyroid dysfunction if not assessed within the last year given chest radiation history.
   - - 1. Would consider TSH to evaluate.

**REFERENCES**

1. Wilson JF. Care of the Adult Cancer Survivor. *Clin*. 2013;6(16).

2. Runowicz CD, Leach CR, Henry NL, et al. American cancer society/American society of clinical oncology breast cancer survivorship care guideline. *J Clin Oncol*. 2016. doi:10.1200/JCO.2015.64.3809.
