# Supplementary material for: A Graduate Medical Education Curriculum to Introduce the Concept of Cancer Survivorship
Source: MedEdPORTAL. 2018 Jan 25;14:10673. doi: 10.15766/mep_2374-8265.10673 (PMC6342428; doi:10.15766/mep_2374-8265.10673)
Supplement: Supplementary file 1 — A. Survivorship Case.docx B. Facilitator Manual.docx C. Pre- and Posttest.docx D. Pre- and Posttest with Answers.docx E. ASCO Survivorship Care Plan Blank.docx F. ASCO Survivorship Care Plan Bonnie Olden.docx [file mep-14-10673-s001.zip › C._Pre-_and_Posttest.docx]

**Pre and Post-Workshop Questionnaire**

1. Who is NOT a cancer survivor?
   1. 45 yo woman just diagnosed with breast cancer
   2. 45 yo woman with history of Hodgkin’s Disease in childhood
   3. 45 yo woman with metastatic breast cancer transitioning to Hospice Care
   4. 45 yo woman with an 8 year old son diagnosed with Hodgkin’s Disease
   5. All the above are cancer survivors.
2. Which of the following is a common long term side effect of cisplatin chemotherapy?
   1. Nausea, vomiting, and diarrhea
   2. Peripheral neuropathy
   3. Diminished cardiac function
   4. Hair Loss
   5. All of the above
3. Which of the following is NOT a common side effect of chest radiation?
   1. Thyroid Dysfunction
   2. Esophagitis
   3. Pericarditis
   4. Pneumonitis
   5. Cognitive Dysfunction
4. Why are there more cancer survivors now than before?
   1. Earlier diagnoses through improved screening
   2. More effective treatments
   3. Prevention of secondary disease and cancer recurrence
   4. Decreases in mortality from other causes
   5. All of the above
5. I feel competent in knowing how to find or create a Survivorship Care Plan.
   1. Strongly Disagree
   2. Disagree
   3. Neutral
   4. Agree
   5. Strongly Agree
6. I feel comfortable in screening for excess mortality in cancer survivors.
   1. Strongly Disagree
   2. Disagree
   3. Neutral
   4. Agree
   5. Strongly Agree
