# Supplementary material for: A Graduate Medical Education Curriculum to Introduce the Concept of Cancer Survivorship
Source: MedEdPORTAL. 2018 Jan 25;14:10673. doi: 10.15766/mep_2374-8265.10673 (PMC6342428; doi:10.15766/mep_2374-8265.10673)
Supplement: Supplementary file 1 — A. Survivorship Case.docx B. Facilitator Manual.docx C. Pre- and Posttest.docx D. Pre- and Posttest with Answers.docx E. ASCO Survivorship Care Plan Blank.docx F. ASCO Survivorship Care Plan Bonnie Olden.docx [file mep-14-10673-s001.zip › D._Pre-_and_Posttest_with_Answers.docx]

**Pre and Post-Workshop Questionnaire with Answers**

1. Who is NOT a cancer survivor?
   1. 45 yo woman just diagnosed with breast cancer
   2. 45 yo woman with history of Hodgkin’s Disease in childhood
   3. 45 yo woman with metastatic breast cancer transitioning to Hospice Care
   4. **45 yo woman with an 8 year old son diagnosed with Hodgkin’s Disease**
   5. All the above are cancer survivors.
2. Which of the following is a common long term side effect of cisplatin chemotherapy?
   1. Nausea, vomiting, and diarrhea
   2. **Peripheral neuropathy**
   3. Diminished cardiac function
   4. Hair Loss
   5. All of the above
3. Which of the following is NOT a common side effect of chest radiation?
   1. Thyroid Dysfunction
   2. Esophagitis
   3. Pericarditis
   4. Pneumonitis
   5. **Cognitive Dysfunction**
4. Why are there more cancer survivors now than before?
   1. Earlier diagnoses through improved screening
   2. More effective treatments
   3. Prevention of secondary disease and cancer recurrence
   4. Decreases in mortality from other causes
   5. **All of the above**
5. I feel competent in knowing how to find or create a Survivorship Care Plan
   1. Strongly Disagree
   2. Disagree
   3. Neutral
   4. Agree
   5. Strongly Agree
6. I feel comfortable in screening for excess mortality in cancer survivors
   1. Strongly Disagree
   2. Disagree
   3. Neutral
   4. Agree
   5. Strongly Agree

**Answer Key and Discussion**

**Question 1**

The definition of a cancer survivor is anyone alive today who has ever received a cancer diagnosis.

Answer choices **A**, **B**, and **C** all fit this definition. While survivorship care does include family members, friends, and caregivers of survivors, the woman described in answer choice **D** does not fit the definition of a cancer survivor.

Correct Answer: **D - 45 yo woman with an 8 year old son diagnosed with Hodgkin’s Disease**

**Question 2**

Side effects from chemotherapeutic agents can generally be divided into short-term, or immediate side effects, and latent side effects. Nausea, vomiting, diarrhea, and hair loss (answer choices **A** and **D**) more commonly occur in the short-term setting, usually during the course of treatment. While diminished cardiac function (choice **C**) can occur in the latent phase, it is not a common side effect of cisplatin therapy. This is more commonly seen with anthracycline therapy, such as doxorubicin.

Peripheral neuropathy is a very common side effect of platinum-based therapies like cisplatin. Since the dorsal root ganglion is not protected by the blood-brain barrier, the DNA within its cell body is preferentially susceptible to toxic agents. The neurotoxicity associated with platinum agents generally presents as a sensory neuropathy with anterograde axonal degeneration. Neuropathy can often develop during the treatment period and its severity usually correlates with treatment dose and duration. The majority of neuropathies resolve after treatment cessation, but there are a significant number of patients who report a progression of sensory loss for many months and sometimes years following completion of therapy.

Correct Answer: **B -** **Peripheral neuropathy**

**Question 3**

Radiation side-effects are generally dependent on the radiation field. In this case, answer choices **A**, **B**, **C**, and **D** are all organs located within the radiation field. In this scenario, the brain is the farthest organ from the radiation field.

Correct Answer: **E - Cognitive Dysfunction**

**Question 4**

Per the American Cancer Society, as of January 2016, it is estimated that there are 15.5 million cancer survivors in the United States, which represents 4.8% of the population. The number of cancer survivors is projected to increase by 31%, to 20.3 million, by 2026. The reason for the growing number of survivors is multifactorial, including improved screening and early detection (answer choice **A**), improved treatment modalities (answer choice **B**), prevention of secondary disease and cancer recurrence (answer choice **C**), and improved treatment of other diseases, mainly cardiac and pulmonary disease, which also contributed to mortality (answer choice **D**).

Correct Answer: **E - All of the above**

**Question 5**

What is a Survivorship Care Plan?

In 2006 the Institute of Medicine issued a report recommending that every cancer patient receive an individualized survivorship care plan that includes guidelines for monitoring and maintaining their health. In response to that report, many groups have now developed various types of "care plans" to help improve the quality of care of survivors as they move beyond their cancer treatment. Care plans may vary based on institution and many institutions have developed their own care plans. The American Society of Clinical Oncology (ASCO) survivorship care plan is just one of the models of care plans available. It is a free and available resource distributed by ASCO and is available here: <https://www.asco.org/practice-guidelines/cancer-care-initiatives/prevention-survivorship/survivorship-compendium>

**Question 6**

What is excess mortality?

Excess mortality refers to causes of death that exist outside of what is expected. With regards to cancer, excess mortality generally includes non-recurrence death specifically related to treatment complications. This would include cardio-pulmonary toxicity from treatment modalities such as chemotherapy and radiation and mortality from secondary malignancies which developed because of treatment exposure.

While cancer recurrence is also a cause of death in survivors, it does not fall under the category of excess mortality as it is a possible outcome related to the diagnosis itself. This is generally classified as *late-recurrence mortality*. Another term, *background mortality*, refers to age-specific mortality in the general population.
