# Supplementary material for: A Graduate Medical Education Curriculum to Introduce the Concept of Cancer Survivorship
Source: MedEdPORTAL. 2018 Jan 25;14:10673. doi: 10.15766/mep_2374-8265.10673 (PMC6342428; doi:10.15766/mep_2374-8265.10673)
Supplement: Supplementary file 1 — A. Survivorship Case.docx B. Facilitator Manual.docx C. Pre- and Posttest.docx D. Pre- and Posttest with Answers.docx E. ASCO Survivorship Care Plan Blank.docx F. ASCO Survivorship Care Plan Bonnie Olden.docx [file mep-14-10673-s001.zip › E._ASCO_Survivorship_Care_Plan_Blank.docx]

| **General Information** | | | | | | | | | |
| --- | --- | --- | --- | --- | --- | --- | --- | --- | --- |
| Patient Name: | | | | | | Patient DOB: | | | |
| Patient phone: | | | | | | Email: | | | |
| **Health Care Providers** (Including Names, Institution) | | | | | | | | | |
| Primary Care Provider: | | | | | | | | | |
| Surgeon: | | | | | | | | | |
| Radiation Oncologist: | | | | | | | | | |
| Medical Oncologist: | | | | | | | | | |
| Other Providers: | | | | | | | | | |
| **Treatment Summary** | | | | | | | | | |
| **Diagnosis** | | | | | | | | | |
| Cancer Type/Location/Histology Subtype: | | | | | | | | | Diagnosis Date (year): |
| Stage: ☐I ☐II ☐III ☐Not applicable | | | | | | | | | |
|  | | | | | | | | | |
| **Treatment** | | | | | | | | | |
| Surgery ☐ Yes ☐No | | | | | Surgery Date(s) (year): | | | | |
| Surgical procedure/location/findings: | | | | | | | | | |
| Radiation ☐ Yes ☐No | Body area treated: | | | | | | | End Date (year): | |
| Systemic Therapy (chemotherapy, hormonal therapy, other) ☐ Yes ☐No | | | | | | | | | |
| Names of Agents Used | | | | | | | | | End Dates (year) |
|  | | | | | | | | |  |
|  | | | | | | | | |  |
|  | | | | | | | | |  |
|  | | | | | | | | |  |
| Persistent symptoms or side effects at completion of treatment: □ No □ Yes (enter type(s)) : | | | | | | | | | |
| **Familial Cancer Risk Assessment** | | | | | | | | | |
| Genetic/hereditary risk factor(s) or predisposing conditions: | | | | | | | | | |
| Genetic counseling: □ Yes □ No Genetic testing results: | | | | | | | | | |
| **Follow-up Care Plan** | | | | | | | | | |
| Need for ongoing (adjuvant) treatment for cancer ☐ Yes ☐ No | | | | | | | | | |
| Additional treatment name | | | | Planned duration | | | Possible Side effects | | |
|  | | | |  | | |  | | |
|  | | | |  | | |  | | |
|  | | | |  | | |  | | |
| **Schedule of clinical visits** | | | | | | | | | |
| Coordinating Provider | | When/How often | | | | | | | |
|  | |  | | | | | | | |
|  | |  | | | | | | | |
|  | |  | | | | | | | |
|  | |  | | | | | | | |
| **Cancer surveillance or other recommended related tests** | | | | | | | | | |
| Coordinating Provider | | | What/When/How Often | | | | | | |
|  | | |  | | | | | | |
|  | | |  | | | | | | |
|  | | |  | | | | | | |
|  | | |  | | | | | | |
| Please continue to see your primary care provider for all general health care recommended for a (man) (woman) your age, including cancer screening tests. Any symptoms should be brought to the attention of your provider:   1. Anything that represents a brand new symptom; 2. Anything that represents a persistent symptom; 3. Anything you are worried about that might be related to the cancer coming back. | | | | | | | | | |
| Possible late- and long-term effects that someone with this type of cancer and treatment may experience: | | | | | | | | | |
| Cancer survivors may experience issues with the areas listed below. If you have any concerns in these or other areas, please speak with your doctors or nurses to find out how you can get help with them.  ☐ Emotional and mental health ☐ Fatigue ☐ Weight changes ☐Stopping smoking  ☐ Physical Functioning ☐ Insurance ☐ School/Work ☐Financial advice or assistance  ☐ Memory or concentration loss ☐ Parenting ☐ Fertility ☐ Sexual functioning  ☐ Other | | | | | | | | | |
| A number of lifestyle/behaviors can affect your ongoing health, including the risk for the cancer coming back or developing another cancer. Discuss these recommendations with your doctor or nurse:  ☐Tobacco use/cessation ☐ Diet  ☐Alcohol use ☐Sun screen use  ☐Weight management (loss/gain) ☐Physical activity | | | | | | | | | |
| Resources you may be interested in: | | | | | | | | | |
| Other comments: | | | | | | | | | |
| Prepared by: Delivered on: | | | | | | | | | |
