# Supplementary material for: A Graduate Medical Education Curriculum to Introduce the Concept of Cancer Survivorship
Source: MedEdPORTAL. 2018 Jan 25;14:10673. doi: 10.15766/mep_2374-8265.10673 (PMC6342428; doi:10.15766/mep_2374-8265.10673)
Supplement: Supplementary file 1 — A. Survivorship Case.docx B. Facilitator Manual.docx C. Pre- and Posttest.docx D. Pre- and Posttest with Answers.docx E. ASCO Survivorship Care Plan Blank.docx F. ASCO Survivorship Care Plan Bonnie Olden.docx [file mep-14-10673-s001.zip › F._ASCO_Survivorship_Care_Plan_Bonnie_Olden.docx]

| **General Information** | | | | | | | | | |
| --- | --- | --- | --- | --- | --- | --- | --- | --- | --- |
| Patient Name: *Bonnie Olden* | | | | | | Patient DOB: *7/20/1944* | | | |
| Patient phone: | | | | | | Email: | | | |
| **Health Care Providers** (Including Names, Institution) | | | | | | | | | |
| Primary Care Provider: *Learner/Facilitator Name, Home Institution* | | | | | | | | | |
| Surgeon: *Elaine Cutting, Home Institution* | | | | | | | | | |
| Radiation Oncologist: *Ex Ray, Home Institution* | | | | | | | | | |
| Medical Oncologist: *Ivy Infu Sion, Home Institution* | | | | | | | | | |
| Other Providers: | | | | | | | | | |
| **Treatment Summary** | | | | | | | | | |
| **Diagnosis** | | | | | | | | | |
| Cancer Type/Location/Histology Subtype:  *LEFT breast adenocarcinoma, 2.5 cm mass with high-grade characteristics. ER positive, HER-2 negative.* | | | | | | | | | Diagnosis Date (year):  *2010* |
| Stage: ☐I ☐II XIII ☐Not applicable | | | | | | | | | |
|  | | | | | | | | | |
| **Treatment** | | | | | | | | | |
| Surgery X Yes ☐No | | | | | Surgery Date(s) (year): *4/29/2010* | | | | |
| Surgical procedure/location/findings:  *LEFT mastectomy and Lymph Node Dissection -- 3/12 lymph nodes contained adenocarcinoma cells*  *Pathology was ER positive and HER-2 negative* | | | | | | | | | |
| Radiation X Yes ☐No | Body area treated: *LEFT Breast* | | | | | | | End Date (year): *8/10/2010* | |
| Systemic Therapy (chemotherapy, hormonal therapy, other) X Yes ☐No | | | | | | | | | |
| Names of Agents Used | | | | | | | | | End Dates (year) |
| *Doxorubicin* | | | | | | | | | *7/10/2010* |
| *Cyclophosphamide* | | | | | | | | | *7/10/2010* |
| *Paclitaxel* | | | | | | | | | *7/10/2010* |
| *Anastrozole (Arimidex)* | | | | | | | | | *current* |
| Persistent symptoms or side effects at completion of treatment: □ No X Yes (enter type(s)) :  *fatigue* | | | | | | | | | |
| **Familial Cancer Risk Assessment** | | | | | | | | | |
| Genetic/hereditary risk factor(s) or predisposing conditions: *None* | | | | | | | | | |
| Genetic counseling: □ Yes □ No Genetic testing results:  *unknown* | | | | | | | | | |
| **Follow-up Care Plan** | | | | | | | | | |
| Need for ongoing (adjuvant) treatment for cancer X Yes ☐ No | | | | | | | | | |
| Additional treatment name | | | | Planned duration | | | Possible Side effects | | |
| *Anastrozole* | | | | *Clarify with Oncology the duration – 5 years or more?* | | | *Fatigue, depression/anxiety, venous thromboembolism, dizziness, confusion, coronary artery disease, hypercholesterolemia* | | |
| **Schedule of clinical visits** | | | | | | | | | |
| Coordinating Provider | | When/How often | | | | | | | |
| *Primary Care Provider* | | *Every 6 months – more frequently if needed* | | | | | | | |
| *Oncologist Ivy Infu Sion* | | *Once a year?* | | | | | | | |
| *Breast Surgeon Elaine Cutting* | | *Once a year? Follow up is operator dependent* | | | | | | | |
| *Radiation Oncologist Ex Ray* | | *Only as needed* | | | | | | | |
| **Cancer surveillance or other recommended related tests** | | | | | | | | | |
| Coordinating Provider | | | What/When/How Often | | | | | | |
| *Primary Care* | | | *Lung Cancer Screening: now and annually per USPSTF guidelines until age 80 given pack year history* | | | | | | |
| *Primary Care* | | | *Cervical Cancer Screening: only if not adequately screened before age 65.* | | | | | | |
| *Primary Care* | | | *Breast Cancer Surveillance: Annual Mammography, annual Medical Oncology visit* | | | | | | |
| *Primary Care* | | | *Chest radiation side effects – check an annual TSH, ask about symptoms of dyspnea on exertion, chest pain, or pain with swallowing and refer to appropriate testing if indicated– echocardiogram, chest xray, or endoscopy respectively.* | | | | | | |
| Please continue to see your primary care provider for all general health care recommended for a (man) (woman) your age, including cancer screening tests. Any symptoms should be brought to the attention of your provider:   1. Anything that represents a brand new symptom; 2. Anything that represents a persistent symptom; 3. Anything you are worried about that might be related to the cancer coming back. | | | | | | | | | |
| Possible late- and long-term effects that someone with this type of cancer and treatment may experience:  *Treatment-based long term effects:*  *Doxorubicin – dyspnea on exertion from underlying cardiomyopathy*  *Cyclophosphamide – secondary cancer is a long term concern with this drug, particularly leukemia -- which can present as fatigue or easy bruising and bleeding-- and bladder cancer – which can present as blood in urine.*  *Paclitaxel – most common long term effect is peripheral neuropathy.*  *Radiation long term effects – consider organs near the radiation field. Given chest radiation, need to consider thyroid, esophagus, lungs, and heart. Symptoms of thyroid dysfunction include fatigue, skin/hair/nail changes, constipation or diarrhea, and palpitations. Symptoms of esophageal dysfunction include difficulty swallowing, reflux, burning, or chest pain. Symptoms of lung dysfunction include dyspnea on exertion and cough. Symptoms of cardiac dysfunction include chest pain, dyspnea on exertion, lower extremity edema, and palpitations.*  *Anastrozole – common concerns are venous thromboembolism, coronary disease, and cognitive dysfunction.* | | | | | | | | | |
| Cancer survivors may experience issues with the areas listed below. If you have any concerns in these or other areas, please speak with your doctors or nurses to find out how you can get help with them.  ☐ Emotional and mental health ☐ Fatigue ☐ Weight changes ☐Stopping smoking  ☐ Physical Functioning ☐ Insurance ☐ School/Work ☐Financial advice or assistance  ☐ Memory or concentration loss ☐ Parenting ☐ Fertility ☐ Sexual functioning  ☐ Other | | | | | | | | | |
| A number of lifestyle/behaviors can affect your ongoing health, including the risk for the cancer coming back or developing another cancer. Discuss these recommendations with your doctor or nurse:  ☐Tobacco use/cessation ☐Diet  ☐Alcohol use ☐Sun screen use  ☐Weight management (loss/gain) ☐Physical activity | | | | | | | | | |
| Resources you may be interested in: | | | | | | | | | |
| Other comments: | | | | | | | | | |
| Prepared by: Delivered on: | | | | | | | | | |
